# EHMTI-0171. Headache disorders - current care of physiotherapy?

**DOI:** 10.1186/1129-2377-15-S1-D18

**Published:** 2014-09-18

**Authors:** A Eder, EJ Hurkmans, C Reindl, L Egretzberger, CH Edthofer

**Affiliations:** 1 University of Applied Sciences, FH Campus Wien, Vienna, Austria

## Introduction

Headache disorders are a major health problem in Europe and lead to widespread suffering and considerable economic consequences. For the treatment of these patients the World Health Organisation recommends the use of management guidelines. In some guidelines beside the treatment with medication other therapies like physiotherapy are also mentioned to be a potential treatment option.

## Aims

So far, it remains unclear, to what extent physiotherapists are nowadays confronted with patients with headache disorders and which physiotherapy interventions are actually applied in this patient population.

## Methods

A cross-sectional study by means of a digital questionnaire was send in January 2013 to 4892 members of Physio Austria (the professional organisation for physiotherapists in Austria). After two weeks a reminder was send.

## Results

The questionnaire was returned by 627 physiotherapists, resulting in a response rate of 13%. 70,1% of this physiotherapists treats in their clinical setting headache patients., either as primary diagnose (70%) or as coexisting symptoms. Interventions like Manual therapy, Relaxation Training or Trigger Point therapy are applied according to clinical findings and anamneses.

## Conclusion

The findings of this study show that physiotherapists are confronted with patients with headache disorders on a regular basis. Therefore it can be recommended that management of headache patients should be included in physiotherapy education and interdisciplinary teams could consider taking physiotherapists on board. Furthermore, scientific studies should focus in effectivity of physiotherapy interventions.

No conflict of interest.

